# Therapeutic Vaccination with Cationic Liposomes Formulated with Dioctadecyldimethylammonium and Trehalose Dibehenate (CAF01) and Peptide P10 Is Protective in Mice Infected with *Paracoccidioides brasiliensis*

**DOI:** 10.3390/jof6040347

**Published:** 2020-12-08

**Authors:** Marcelo Valdemir de Araújo, Samuel Rodrigues Dos Santos Júnior, Joshua D. Nosanchuk, Carlos Pelleschi Taborda

**Affiliations:** 1 Departamento de Microbiologia, Instituto de Ciências Biomédicas, Universidade de São Paulo, São Paulo 05508-000, Brazil; marceloaraujo@usp.br (M.V.d.A.); samuelmicrobio@usp.br (S.R.D.S.J.); 2 Departments of Medicine (Division of Infectious Disease), Microbiology and Immunology, Albert Einstein College of Medicine and Montefiore Medical Center, Bronx, NY 10461, USA; nosanchuk@gmail.com; 3 Departamento de Dermatologia, Instituto de Medicina Tropical de São Paulo—LIM53, Faculdade de Medicina, Universidade de São Paulo, São Paulo 4023-062, Brazil

**Keywords:** paracoccidioidomycosis, *P. brasiliensis*, peptide vaccine, adjuvant, CAF01, DDA/TDB, cationic liposome

## Abstract

The peptide P10 is a vaccine candidate for Paracoccidioidomycosis, a systemic mycosis caused by fungal species of the genus *Paracoccidioides* spp. We have previously shown that peptide P10 vaccination, in the presence of several different adjuvants, induced a protective cellular immune response mediated by CD4^+^ Th_1_ lymphocytes that was associated with the increased production of IFN-γ in mice challenged with a virulent isolate of *Paracoccidoides brasiliensis*. Cationic liposomes formulated with dioctadecyldimethylammonium and trehalose dibehenate (DDA/TDB, termed also CAF01–cationic adjuvant formulation) have been developed for safe administration in humans and CAF01 liposomes are utilized as an adjuvant for modulating a robust Th_1_/Th_17_ cellular response. We evaluated the efficacy of the adsorption of peptide P10 to CAF01 cationic liposomes and used the generated liposomes to vaccinate C57Bl/6 mice infected with *P. brasiliensis*. Our results showed that P10 was efficiently adsorbed onto CAF01 liposomes. The vaccination of infected mice with cationic liposomes formulated with DDA/TDB 250/50 µg/mL and 20 µg of P10 induced an effective cellular immune response with increased levels of Th_17_ cytokines, which correlated with significant decreases in the fungal burdens in lungs and protective granulomatous tissue responses. Hence, cationic liposomes of DDA/TDB 250/50 µg/mL with 20 µg of P10 are a promising therapeutic for safely and effectively improving the treatment of paracoccidioidomycosis.

## 1. Introduction

Paracoccidioidomycosis (PCM) is a systemic fungal disease that is due to infection by the thermally-dimorphic fungi of the genus *Paracoccidioides* [1]. These pathogens are found in soil as saprophyte mycelium [1]. Disturbances in the environment can aerosolize hyphal fragments or conidia, which can be inhaled and subsequently deposited in the alveoli where they undergo morphogenic transformation to a yeast phase [2]. *Paracoccidioides brasiliensis* was for many years considered as the only species in the genus [3] until Teixeira et al. (2014) described a second species, known as *P. lutzii* [4]. However, further detailed molecular studies identified that there are five phylogenetic species in the genus *Paracoccidioides*: *P. lutzii* and four cryptic species of the *P. brasiliensis*: S1, PS2, PS3, and PS4 [5,6,7,8]. The formerly aggregated *P. brasileinsis* species are currently referred to as *P. brasiliensis* (S1), *P. americana* (PS2), *P. restrepensis* (PS3), *P. venezuelensis* (PS4), and *P. lutzii* [3].

PCM principally occurs from the south of Mexico to the north of Argentina, and it is considered one of the most important systemic fungal infections in the geographic region that corresponds to Latin America [1]. Antifungal treatment is essential for achieving a cure and durations of therapy are prolonged, frequently requiring 2 or more years of therapy [2,9]. Two clinical forms are well known. The acute form (juvenile type) is the more aggressive, frequently systemic manifestation, which affects children, adolescents, and young adults (30 to 40 years) and is homogenous between genders [2]. The chronic form (adult type) is significantly more common (74% to 96% of PCM cases) and affects adults between 30 a 60 years of age with males being more commonly afflicted [2].

An efficient cellular immune response is crucial in host defense against fungal pathogens such as *Paracoccidioides* spp. [10]. Therapeutic or prophylactic vaccines are important promising tools for the prevention or treatment of patients with fungal infections [11]. The peptide P10 (QTLIAIHTLAIRYAN) has been highlighted as a vaccine candidate against PCM [12]. Derived from a *P. brasiliensis* glycoprotein of 43kDa (gp43) [13], P10 is considered the main diagnostic antigen [14,15]. Although the orthology of a glycoprotein of 43kDa in *P. lutzii* (Plgp43) has only 80% of identity with gp43, both are structurally related to fungal exo-glucanases [16]. Previous studies have shown that the administration of P10 vaccine with different adjuvants can reduce the fungal burden and elicit a mixed cellular immune response characterized by a predominant Th_1_ response with production of IFN-γ, TNF-α and IL-12 in murine infection models [11,17].

In general, peptides alone are poorly immunogenic and require adjuvants and delivery systems to be effective [18]. The use of an adjuvant combined with a specific antigen produces a more robust immune response in experimental PCM compared to the antigen alone [10]. Cationic liposomes can potentiate subunit vaccines in addition to helping to decrease the vaccine dose required for efficacy [19]. The amphiphilic synthetic lipid DODAB (dioctadecyldimethylammonium bromide) is a surfactant-based in ammonium quaternary (DDA), which can function as a cationic liposome that can be used as a carrier in a drug delivery system and as an adjuvant [20].

The level and quality of an immune response induced by DDA liposomes may be enhanced by the incorporation of an immunostimulatory compound, such as trehalose dibehenate (TDB) [21], a synthetic analogue of trehalose dimycolate (TDM), known as a cord factor. However, it is considered unacceptable for clinical use because it is an important factor in the granulomatous response of mycobacteria [21,22,23,24]. Therefore, TDB was modified by replacing long branched mycolic acid (>70 carbons) [25] for two long 22-carbon acyl chains (behenic acid) [21] resulting in a compound with lower toxicity that retains its adjuvant activity [26,27]. The modified TBD acts on the Mincle receptor by activating Syk-Card9 signaling in antigen-presenting cells (APCs) [25,28,29,30].

The combination of DDA with TDB results in the adjuvant known as CAF01 (*cationic adjuvant formulation)*, which induces a potent immune response with production of high level with IFN-γ, IL-17, and low levels of IL-5 [21,31]. CAF01 has been tested in phase I clinical trial in humans volunteers against tuberculosis in combination with Ag85B and ESAT 6 (H1) antigen (H1:CAF01) [32].

In the current study, we have adsorbed the *P. brasiliensis* peptide P10 onto DDA/TDB adjuvant (CAF01). Our data shows that P10 with DDA/TDB is a promising adjuvant-boosted vaccine as it efficiently decreased fungal lung burden in C57Bl/6 mice infected with *P. brasiliensis* in a therapeutic study. Additionally, the vaccine increased the levels of pro-inflammatory cytokines, such as IL-17, in mice with PCM.

## 2. Materials and Methods

### 2.1. Animals

We obtained 6-to 8-week-old male C57BL/6, weighing between 25 to 30 g, from the Animal facility at Faculdade de Medicina da Universidade de São Paulo under Specific-Pathogen-Free conditions and transferred to the animal facility at Departamento de Microbiologia do Instituto de Ciências Biomédicas da Universidade de São Paulo. All procedures were performed according to the guidelines of National Council of Ethics with Animals (CONCEA) and the protocols were approved by the Ethical Committee for Animal Use from Institute of Biomedical Sciences at University of Sao Paulo (CEUA ICB USP certificates 101/2014, approved in 01/12/2014).

### 2.2. Peptide P10 Preparation

The peptide P10 (QTLIAIHTLAIRYAN) [12] was synthesized and purified by Aminotech (São Paulo, SP, Brazil) with a purity grade of >94% as confirmed by mass spectrometry and HPLC. The stock solution (1000 µg/mL) was prepared by adding 20% DMSO (Dimethyl sulfoxide) Sigma (St. Louis, MO, USA) and 80% 1 mM Tris-buffer (Carlsbad, CA, USA). The stock was aliquoted and stored in the freezer −20 °C until use. The peptide was thawed and just added to liposomes, described below, with an interaction time of 1 h at 25 °C [33].

The Mincle agonist D-(+) trehalose 6,6′-dibehenate (TDB) was obtained as a powder from Avanti Polar Lipids (Sigma, Alabaster, AL, USA) and stored at −20 °C according to manufacturer’s instructions. The specified quantities were weighed at the time of preparation of liposomes and added to DDA. The cationic lipid dioctadecyldimethylammonium bromide (DODAB) was purchased from Sigma (St. Louis, MO, USA), at >98% purity as confirmed by TLC.

### 2.3. Preparation of Liposome

Unilamellar liposomes of DDA/TDB were prepared by the film hydration method [34,35]. The method consists of dilution of DDA/TDB (5:1) (1.25/0.5 mg/mL) in a mixed solution of chloroform/methanol (9:1). The removal of organic solvent was achieved by a rotary evaporator and the process was stopped when a film was observed on the bottom of the bottle. The liposomes were hydrated with 10 mM tris-buffer for 20 min at a temperature 10 °C above transition phase (*T_m_* = 47 °C) until complete hydration occurred and then the liposomes were stored at 4 °C for up two weeks [35].

### 2.4. Determination of Diameter Size, Polydispersity and Zeta Potential of Liposomes

The diameters (Dz), polydispersity index (Pdi), and zeta potential (ζ) of the liposomes were determined using a Zetasizer (Nano ZS Malvern Instruments, Worcestershire, UK). The samples were dispersed with 1mM tris-buffer (pH: 7.4) with a dilution rate of 1:300. The refractive index of pure water (1.0) was used as a baseline [35,36].

### 2.5. Adsorption Efficiency

The adsorption efficiency of liposomes and antigen was estimated by the following Equation (1) [37,38]:

(1) $$\mathrm{EE}\left(\%\right)=\frac{\mathrm{Total}\;\mathrm{Antigen}\;-\;\mathrm{Free}\;\mathrm{Antigen}}{\mathrm{Total}\;\mathrm{Antigen}}\times 100\%$$

Free antigen was separated from liposomes by ultracentrifugation and quantified by Qubit protein assay (Invitrogen, Eugene, OR, USA). For separation, the samples (liposome plus peptide) were centrifuged at 36,000 rpm (100,000× *g*, Ultracentrifuge Beckman Coulter Optima XL-100K, rotor type 70.1Ti) for 60 min, 4 °C, and then the supernatant with free antigen was collected. The measurements were performed using 20 µL of samples in 180 µL of working solution (kit Qubit) incubated for 15 min and read on a Qubit fluorometer.

### 2.6. Determination of Liposomes Morphology by Transmission Electronic Microscopy (TEM)

The morphological analysis of unilamellar DDA/TDB liposomes was performed using a Jeol 1200EX transmission electron microscope with LaB6 filament operating at 80 kV voltage. For measurements, liposomes samples were diluted in 1 mM tris-buffer (pH: 7.4), 10 µL were placed on a copper grid and dried at room temperature (approximately 2 min), then the excess was removed with a paper filter and 10 µL of Uranyl was added. The mixture remained at room temperature for approximately 5 min, the excess was withdrawn with a paper filter [35].

### 2.7. Experimental Infection

The well-characterized, virulent isolate *P. brasiliensis* (Pb18) [39] was used to infect mice via intratracheal injection. The isolate was maintained by weekly passages on Fava Netto solid medium at 37 °C. After 7 to 10 days of growth, yeast cells were transferred to Brain Heart Infusion medium (Becton and Dickinson, Sparks, MD, USA) supplement with 4% fetal bovine serum (Gibco, Grand Island, NY, USA) and gentamicin 50,000 mg (Gibco, Grand Island, NY, USA) (40 µg/mL) and then incubated with rotary shaking at 37 °C for 7 days. Yeast cells were collected, washed with Phosphate Buffered Saline (PBS), pH: 7.2, and passed through a 1 mL syringe attached to a 26-gauge hypodermic needle to dissociate clustered cells. The cell concentration was determined by counting using a Neubauer’s chamber. Viability was determined by Trypan blue (Sigma, St. Louis, MO, USA) staining and was always higher than 90%. C57Bl/6 mice were intraperitoneally anesthetized with 300 µL of a solution of Xylazine 2 g/100 mL and Ketamine 10 g/mL in PBS buffer (both from União Química Farmacêutica, São Paulo, Brazil). Then, the mice had their tracheas exposed for injection with a 50 µL of a solution containing 3 × 10^5^ yeasts of Pb18. The incisions were sutured with 4-0 silk, and the animals were rested until they recovered from the procedure.

### 2.8. Vaccination Protocols

Different concentrations of DDA/TDB liposome (250/50 µg/mL, 312.5/62.5 µg/mL and 500/100 µg/mL) were combined with 20 µg of peptide P10. The mixtures were kept for 20 min at room temperature. Five groups of mice (each one with 6 animals) received the vaccine formulation or control solutions (DDA/TDB alone). The animals received the first vaccination subcutaneously with 100 µL of vaccine or controls solutions at the base of the tail 30 days after infection. In total, mice received 3 doses at 2 weeks intervals between them. Two weeks after the last vaccination, the animals were euthanized, and their lungs were excised and analyzed for fungal burden, histology, and levels of cytokines.

### 2.9. Determination of Fungal Burden

After euthanasia of mice, the lungs were excised and weighed immediately. The tissues were then manually homogenized in PBS buffer, adjusting to a volume of 2 mL. A portion of homogenate was plated in solid BHI medium, supplemented with 5% culture filtrate of *P. brasiliensis* isolate 192, plus 4% inactivated fetal bovine serum (Gibco, Grand Island, NY, USA), 1% streptomycin and penicillin (Sigma, St. Louis, Mo, USA). The plates were maintained at 37 °C for a period of 7 to 15 days. The number of Colony Forming Units (CFUs) was counted, and results were expressed per gram of tissue.

### 2.10. Quantification of Cytokines Levels of Homogenate Pulmonary for the ELISA Method

Cytokines were determined in the supernatants from lung homogenates by enzyme-linked immunosorbent assay (ELISA) as described [40]. The levels interleukin-4 (IL-4), interleukin-12 (IL-12), interferon-gamma (IFN-γ), interleukin-6 (IL-6), interleukin-10 (IL-10), and tumor necrosis factor-alpha (TNF-α) were determined using ELISA kits (BD Biosciences, San Diego, CA, USA). Interleukin-17 (IL-17) was measured using Biolegend’s ELISA Max (San Diego, CA, USA).

### 2.11. Histopathological Lungs Analysis

A fraction of lung tissue was collected and fixed in formalin 10% (Merck, Darmstadt, Germany). The fragments were embedded in paraffin, and 4 to 5 µm sections were cut and stained with hematoxylin-eosin (H.E.). The images were acquired with an inverted microscope (Zeiss, Primovert, Gottingen, Germany) coupled to a digital camera system (Axiocam 105 color, Zeiss, Oberkochen, Germany) and processed by the Zeiss Software (Zen core, Oberkochen, Germany) in the Laboratory of the immunobiology of interaction Leishmania-macrophages of Instituto de Ciências Biomédicas da Universidade de São Paulo.

### 2.12. Statistical Analysis

The results were analyzed using GraphPad Prism 5.0 Software GraphPad Inc. (San Diego, CA, USA) and analysis of variance (ANOVA) was performed followed by the Bonferroni post-test. The results were considered significant when *p* < 0.05.

## 3. Results

### 3.1. The Effect of Peptide P10 Adsorption on DDA/TDB Liposomes

The physicochemical characteristics of DDA/TDB and DDA/TDB/P10 liposomes prepared by the film hydration method are demonstrated in Table 1. The liposomes alone (DDA/TDB) were dispersed in 1 mM Tris buffer and presented an average diameter size of 471.5 ± 2 nm. The stability of the suspension was maintained by the positive surface charge of liposomes (44.3 ± 2 mV). The polydispersity index (0.528) of the DDA/TBD liposomes is considered average and within pre-established parameters [41]. The adsorption of the peptide onto liposome was accomplished by the simple addition of peptide P10 into the solution for approximately 20 min at room temperature (25 °C). The adsorption of the peptide modified the diameter size of the liposome, increasing it to 667.9 ± 3 nm; furthermore, the Pdi value decreased to 0.302, this value is considered the better dispersity for liposomes, indicating a homogenous population of phospholipid liposomes [42,43,44,45] and the ζ potential of liposomes was 39 ± 3 mV. Interestingly, the changes in the concentrations of DDA/TDB in the formation of liposomes only modestly changed the diameter size, Pdi, or ζ potential, as shown in Table 1.

### 3.2. Determination of Liposomes Morphology after P10 Adsorption by TEM

**Figure 1:**
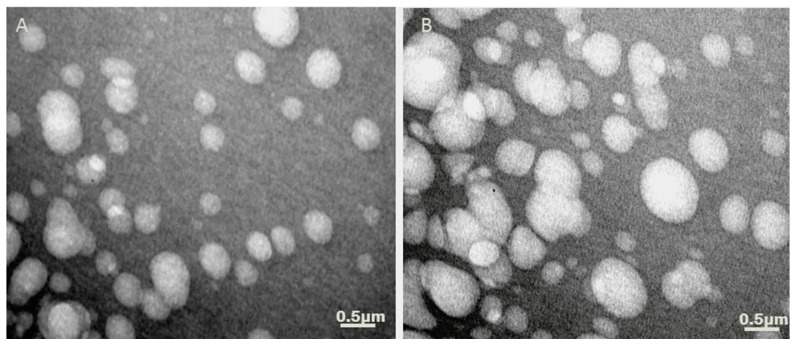
TEM of cationic liposomes. The liposomes were prepared by the film hydration method and fixed with Uranyl for morphological analyses. The micrographs show unilamellar liposomes of DDA/TDB alone (**A**); DDA/TDB plus P10 (**B**).

We used transmission electron microscopy (TEM) to investigate the structure of the liposome and check for possible changes in the liposome after the adsorption of the peptide to it. As shown in Figure 1, the liposomes formed by the film hydration method presented spherical with several sizes, and without signs of aggregation. The adsorption of peptide onto liposomes did not markedly modify its morphology, the liposomes plus peptide demonstrated spherical morphology resembling as liposome alone.

### 3.3. Adsorption of Peptide to Adjuvant Liposome

After the formation of the liposomes, the antigen was added to the structure and then the efficiency of P10 adsorption was evaluated by separating the free antigen by ultracentrifugation. As seen in Table 2, the peptide was efficiently adsorbed onto the liposome at a rate of 80%. The efficiency is consistent with the increase in the liposome/peptide diameter (667.9 nm) compared to the liposome alone (471.5 nm), indicating the accumulation of peptide.

### 3.4. DDA/TDB/P10 Vaccination Controls Pulmonary Fungal Burden

**Figure 2:**
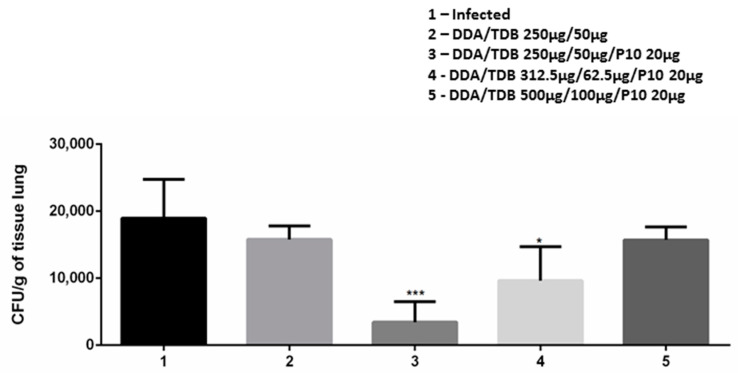
Vaccination with DDA/TDB/P10 decreases the fungal load. The fungal burden was measured in the lungs of mice infected with *P. brasiliensis* via Colony Forming Units (CFU) assay and the results were expressed as CFU/g of lung tissue. Infected animals either received PBS (1), DDA/TDB alone (2), or different formulations of DDA/TBD with P10 (3, 4, and 5). The data represent the mean and SD of results from 3 experiments using 6 mice per group. An asterisk (*) represents a statistically significant difference, *** *p* < 0.001 and * *p* < 0.05.

The immunogenic effect of the different concentrations of DDA/TDB/P10 liposomes was evaluated two weeks after the last of third vaccination. The CFU from the lungs of infected mice is shown in Figure 2. The vaccination with DDA/TDB/P10 in the concentration of 250/50 µg/mL plus 20 µg of P10 showed the best effect with a significant reduction of fungal burden in the lungs (*p* < 0.001), which is a decrease of about 15,000 CFU/g of tissue. This reduction was less pronounced in the formulation of 312.5/62.5 µg/mL with P10 (*p* < 0.05), and DDA/TDB 500/100 µg/mL with P10 did not significantly alter the fungal burden (Figure 2).

### 3.5. The Therapeutic Effect of DDA/TDB/P10 Vaccination Correlates with an IL-4/IL-17 Balance in the Lung Parenchyma

**Figure 3:**
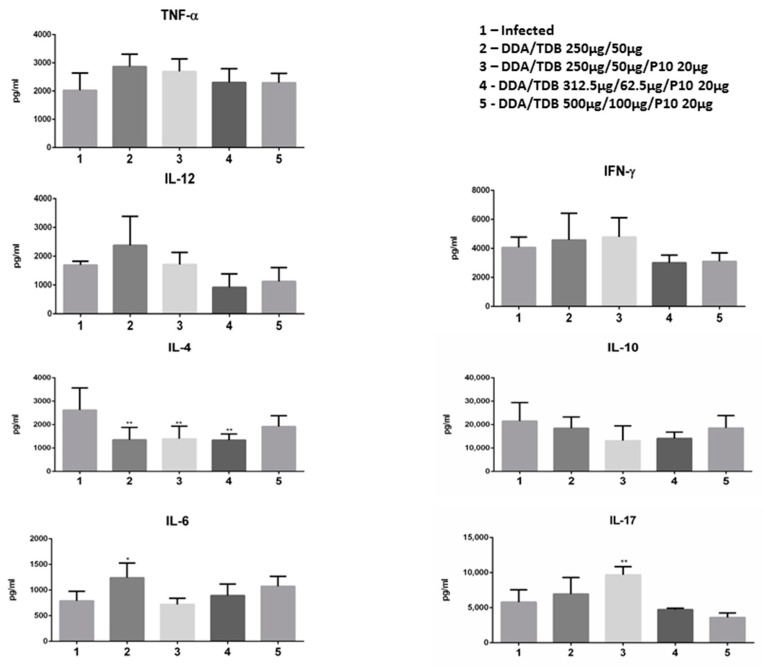
Evaluation of immune responses induced by P10 peptide adsorbed on DDA/TDB liposomes. Th_1_, Th_2_, and Th_17_-associated cytokines were measured in pulmonary homogenates 75 days after infection by capture enzyme-linked immunosorbent assay (ELISA). Infected mice with *P. brasiliensis* received three doses of either PBS as a control (1), DDA/TDB alone (2), or different concentrations of DDA/TDB with P10 (3–5). The data are shown are the mean and SD of results from three independent experiments using 6 mice per group. The asterisk represents a statistically significant difference * *p* < 0.05 and ** *p* < 0.01.

We evaluated whether the adsorption of the peptide onto the liposome altered the profile of cytokines in infected and immunized animals. For this, the cytokine levels corresponding to the Th_1,_ Th_2_, and Th_17_ immune responses of the pulmonary homogenate were evaluated by the ELISA method and the results are shown in Figure 3. As seen in the graphs, the therapeutic vaccination with DDA/TDB (250/50 µg/mL) plus P10 (20 µg) or liposome alone (250/50 µg/mL) in the *P. brasiliensis* infected mice, did not induce alterations of levels of cytokine associated with the Th_1_ immune response. On the other hand, vaccination with the liposome alone, or peptide plus liposome, and with lower concentrations (250/50 and 312.5/62.5 µg/mL) of the liposome, led to decreased levels of IL-4 (*p* < 0.01); a cytokine associated with a Th_2_-biased response. The induction of the Th_17_ response in therapeutic vaccination with liposome associated P10 was also assessed in the pulmonary homogenate (Figure 3). The Th_17_ signature cytokine, IL-17A, had higher levels only following vaccination performed with the formulation of the peptide P10 adsorbed to the liposome in low concentration (250/50 µg/mL) (*p* < 0.01). Notably, the levels of cytokine IL-6, associated with Th_17_ polarization, increased only in mice treated with liposome alone (*p* < 0.05).

### 3.6. Lung Histology

**Figure 4:**
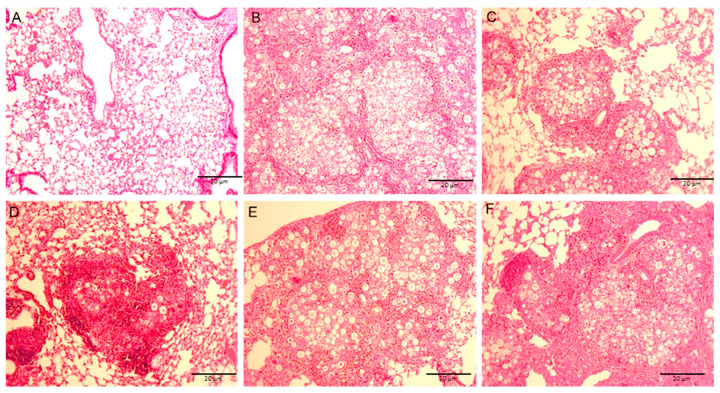
Photomicrographs of pulmonary tissues from C57BL/6 mice with or without *P. brasiliensis* infection. The lung sections were stained with Hematoxylin and Eosin to analyze the presence of inflammation. Uninfected lung tissue (**A**) is presented for comparison with *P. brasiliensis* infected lungs from mice that received PBS only (**B**), treated with DDA/TDB alone (**C**), or vaccinated with DDA: DDA/TDB 250/50 μg/P10 20 μg (**D**) DDA/TDB 312.5/62.5 μg/P10 20 μg (**E**) or DDA/TDB 500/100 μg/P10 20 μg (**F**). (10× magnification).

We performed a histological analysis of lung tissue of all animals included in our protocol using hematoxylin-eosin staining to assess for the presence of granulomas and yeast cells (Figure 4). Lungs from mice infected, but untreated, showed a large number of yeast cells that were diffusely distributed and there was an absence of an organized cellular response (Figure 4B). Animals infected that received DDA/TDB developed well-delimited granulomas containing yeast cells (Figure 4C). Mice infected with *Paracoccidioides* and vaccinated with DDA/TDB 250/50 μg/P10-20 μg showed more compact granuloma with yeast cells inside and the granulomas were surrounded by a dense mass formed by inflammatory cells. Notably, the parenchyma adjacent to the granulomas appeared normal and there were no disseminated yeasts (Figure 4D). The two other DDA/TDB and P10 formulations also induced granuloma formation; however, there was an increased number of yeast cells inside the granulomas and a less dense adjacent infiltration of immune cells (Figure 4E,F).

## 4. Discussion

In this study, we evaluated the immunomodulatory response of the P10 peptide adsorbed onto unilamellar liposome formed by the DDA/TDB adjuvant (CAF01) in an experimental PCM model using C57BL/6 mice infected with *P. brasiliensis* (Pb18). We previously published that other adjuvants, such as CFA (complete Freund’s Adjuvant), associated with peptide P10 significantly reduced the fungal burden of mice infected with *P. brasiliensis* [46]. We also previously showed that dioctadecyldimethylammonium bromide (DODAB) associated with peptide P10 efficiently reduced lung fungal burden of mice infected with *P. brasiliensis* [17]. However, we have continued to seek an effective adjuvant that is deemed safe in humans.

The Cationic Adjuvant Formulation (CAF01 comprised by DDA/TDB) was developed as a safe adjuvant for humans and animals and its mechanism of action is through the triggering of Th_1_ responses [31]. CAF adjuvant has been tested in either human or animals infections with Chikungunya vírus [47], Influenza [48], *Chlamydia trachomatis* [49], *Mycobacterium tuberculosis* [50,51], *Plasmodium falciparum* [52], and *Streptococcus pyogenes* [53].

PCM requires prolonged antifungal treatment, with durations frequently extending past 2 years. This protracted therapy often leads to discontinuation of medications by the patient. Yet, even after prolonged treatment, relapses may occur [54]. Strategies that combine the use of antifungal drugs and therapeutic vaccines may help reduce treatment time, effective recovery of the immune system, and prevent sequelae, including relapses [54]. The ability of antifungal drugs combined with peptide P10 to improve outcomes in experimental PCM has been effectively demonstrated [54]. Hence, there is a solid scientific basis and an important clinical need for a therapeutic PCM vaccine based on P10.

The adsorption of P10 onto liposomes of DDA/TDB modified the physicochemical characteristics of adjuvant. The surface charge remained positive and higher than 30mV, which is essential for the repulsion between liposomes to minimize the likelihood of aggregation [18]. Notably, the adsorption of P10 onto the liposomes changed the diameter size of liposomes from 471.5 nm to 667.9 nm. The size of a liposome may affect the development of specific immune responses, driving the cytokine profile towards a Th_1_ or Th_2_ response [26,55,56].

In our experiments, we observed that the concentration of DDA/TDB was critical for the efficiency of the vaccine preparation in modifying the pathobiology of experimental PCM. We tested three different formulations of DDA/TDB with 20 μg of peptide P10, which was the amount of peptide previously standardized by our group as an effective protective dose of P10 [12,46]. The preparations of liposomes with DDA/TDB 250/50 µg/mL and 312.5/62.5 µg/mL, but not 500/100 µg/mL, were able to significantly reduce pulmonary fungal burdens when the vaccine was administered to infected mice. However, DDA/TDB 250/50 µg/mL with P10-20 µg was significantly more efficient compared to the DDA/TDB 312.5/62.5 µg/mL and 500/100 µg/mL formulations.

The choice of these concentrations was based on the work of Van Dissel 2014 [32], which utilized different concentrations of DDA/TDB in a human trial evaluating the efficiency of prophylactic vaccination against tuberculosis with H1 protein. In their study, Van Dissel and collaborators showed that intermediate and higher concentrations of liposomes induced T cell memory, and this profile was responsive until 150 weeks after vaccination [32]. In our work, low concentrations of DDA/TDB significantly modulated protective immune response with P10, resulting in a significant decrease of burden fungal, an increase of cytokines Th_17_-associated, and the formation of compact granulomas. Curiously, the intermediate and higher concentrations of liposomes did not modulate the immune response against fungal infection.

Our first hypothesis focused on the physical-chemical characteristics of liposomes, with intermediate and higher concentrations, whether these characteristics could increase responsiveness of the cells of the immune system. However, the analysis of diameter size and Pdi are associated with efficacy of liposomes and delivery systems. For better uptake of liposomes by antigen-presenting cells (APCs), the size of the liposome can vary between 500 to 1000 nm [57], in our study all the liposomes were approximately 650 nm (Table 1). The Pdi of liposomes indicated values within of parameters between 0.3 and 0.4. For a population to be considered homogeneous, Pdi values of 0.3 or less are required [41,43,44,45].

Then, we formulated the hypothesis that the amount of peptide in these formulations might not be enough to prime the dendritic cells and modulate the immune response. The rate of phagocytosis is dependent on the concentration of the liposome [58]. A study carried out by Allen 1991 [59] with mouse bone marrow-derived macrophages demonstrated that the uptake of liposomes by macrophages is greater with higher concentrations of the liposome [58,59]. These data were corroborated by Bose 2015 [60], who studied the influence of cationic lipid concentration (DOTAP) on the formation of nanospheres, and their uptake in vitro by HeLa cells, the increase in DOTAP concentration (from 6% to 24%) revealed a higher uptake rate cells (>85%). We hypothesize these concentrations of liposomes, intermediate and higher, of DDA/TDB, could require higher amounts of antigen to modulate immune response similar to the low concentration of liposome. As mentioned above, the amount of peptide used in this work was based on other works of our group [12,46] and indicated that 20 µg of the peptide was sufficient to elicit a protective immune response against PCM in those conditions. So, we hypothesize that lower concentrations of cationic liposome such as 62.5/25 µg/mL with 20 µg of P10, or less, could also modulate a protective immune response.

Once the liposomes of higher concentrations of DDA/TDB did not helped in the modulation of immune response, we prioritize the DDA/TDB 250/50 µg/mL formulation to investigate the effect of the adsorption of peptide to liposome by adsorption efficiency. The P10 peptide was efficiently adsorbed onto DDA/TDB 250/50 µg/mL liposome with a rate higher than 80%. It is important to remember that the degree of adsorption depends on electrostatic forces between components (antigen and liposome). Antigens with an isoelectric point (pI) below 7.4 have a higher rate of adsorption when compared with protein antigens with pI above 7.4 [61,62,63]. The cationic characteristic of P10 peptide (pI = 9.95) (isoelectric.org) could lead to difficulties with adsorption onto liposomes. However, the process of adsorption can be influenced by other features besides pI, like distribution of charge and flexibility of antigen, pH, ionic forces e composition of buffer [61].

Interestingly, the association of the P10 to the DDA/TDB induced an increase in the levels of IL-17A cytokine, while the levels of IL-6 cytokine did not change. However, the level of IL-6 cytokine was significantly increased in the mice group vaccinated with liposome alone. The cytokine IL-17A has the function of inducing granulopoiesis, inflammatory response, recruiting neutrophils, inducing fungicidal activity, inducing microbial peptides such as S100A7, S100A8 [64,65,66,67], and promotion of resistance to infection [68,69,70]. The Th_17_ response induced by vaccination has been pointed out as particularly positively impactful in several studies with vaccine candidates against fungal infection. The importance of the Th_17_ response has been demonstrated in a study of vaccination against three species of dimorphic fungi (*Coccidioides posadasii*, *Histoplasma capsulatum*, and *Blastomyces dermatitidis*) [71]. The Th_17_ response was evaluated by neutralization of IL-17A with monoclonal antibodies, blocking of IL-17A with adenovirus overexpressing IL-17R: soluble Fc, and vaccination of knockout mice for the receptor by IL-17A (IL-17AR). In all experiments, the animals failed to generate resistance against infection by dimorphic fungi [71], underscoring the importance of the Th_17_ response. In our study, the protective antifungal response induced by IL-17A led to a decrease in the fungal load of mice vaccinated with P10 plus liposome, probably due to the increased inflammatory cells demonstrated by lung histology.

Many cell types can produce IL-6 [72,73], and the association of IL-6 plus TGF-β cytokines is important for the induction of transcription factor RORγ-t, which induces the differentiation of Th_17_ cells [72,74,75]. Indeed, DDA/TDB induces potent CD4 Th_1_ and Th_17_ responses [31]. In this work, the rise of levels of cytokine IL-17 occurred without the concomitant rise of IL-6. One hypothesis is that the production of IL-6 cytokine may have occurred at earlier events or may have been consumed during Th17 differentiation.

The reduction in the fungal load in the lungs of animals infected with *P. brasiliensis* and vaccinated with DDA/TDB/P10 was consistent with the increase in the level of IL-17A cytokine and decrease in IL-4 level. In tuberculosis and PCM, an effective CD4 T cell response was essential to control the diseases [76,77]. A prophylactic vaccine (started after 30 days of mice infection) with P10 in the presence of Complete Freund’s Adjuvant in combination with simultaneous treated with Trimethoprim-Sulfamethoxazole enhanced the efficacy of vaccination [46]. Also, the discontinuation of drug treatment in the group of vaccinated mice demonstrated that the immunological state of the mice was effective in preventing relapses [46]. In our current work, we show that DDA/TDB 250/50 μg/P10-20 μg generates an immunological response that leads to a significant reduction in the pulmonary fungal load, which did not occur in animals that received DDA/TDB only.

Granulomatous lesions are important structures in host defense against fungi [78]. This immune reaction functions to restrict the spread of the pathogen and this protective innate immune response is impaired in several forms of immune deficiencies such as HIV or due to drugs like prednisone [79]. Overall, our data are in agreement with studies carried out with knockout animals for IL-6 and IL-17 cytokines in an experimental PCM model, where it was demonstrated that the absence of these cytokines led to the formation of loose and poorly structured granulomas [78]. Moreover, the number of viable fungal cells in granulomas increased with increasing liposome concentrations, highlighting the failure to generate a protective immune response in these groups.

In our study, infected and untreated mice did not effectively form granuloma, whereas mice vaccinated with DDA/TDB alone or DDA/TDB 250/50 µg/mL with P10-20 µg developed well-delimited granuloma containing yeast cells. In particular, vaccination with DDA/TDB/P10 led to the formation of well structured, compact granuloma with the presence of cell infiltrates, showing that treatment was effective in producing a satisfactory response, and recruitment of cells of immune system to infection site.

## 5. Conclusions

Overall, our data suggest that the use of P10 peptide adsorbed onto the cationic liposome DDA/TDB (CAF01) maintains the immunomodulatory properties of DDA/TDB and the DDA/TDB 250/50 μg/P10-20 μg therapeutic vaccine markedly enhances the antifungal potency of the host response against *P. brasiliensis.* These data support ongoing efforts to translate a P10 vaccine from the bench to the bedside.

## Figures and Tables

**Table 1:** Characterization of liposomal liposomes.

Description	Size (nm)	Polydispersity Index	Zeta Potential (mV)
DDA /TDB 250/50 μg	471.5 ± 2	0.528	44.3 ± 2
DDA/TDB 250/50 μg/P10 20 μg	667.9 ± 3	0.302	39 ± 3
DDA/TDB 312.5/62.5 μg/P10 20 μg	650.8 ± 5	0.369	44.7 ± 3
DDA/TDB 100/500 μg/P10 20 μg	639.2 ± 3	0.411	45.7 ± 4

Measurements of diameter size (Dz), polydispersity index (Pdi) and zeta potential (ζ). Results denote mean ± S.D. from 3 different analysis.

**Table 2:** Adsorption efficiency of peptide P10 onto liposome of DDA/TDB.

Description	Adsorption Efficiency %
DDA/TDB 250/50 μg/P10 20 μg	84.5% (*)

*: The data represent three independent measurements. Protein (peptide) concentration refers to the concentration of peptide initially added to the liposome before the ultra-centrifugation process, and the final concentration refers to the amount of free peptide in the supernatant. These results represent the calculations performed according to the instructions of the Qubit kit manufacturer.
